# Film Coating of Nifedipine Extended Release Pellets in a Fluid Bed Coater with a Wurster Insert

**DOI:** 10.1155/2014/520758

**Published:** 2014-03-18

**Authors:** Luciane Franquelin Gomes de Souza, Marcello Nitz, Osvaldir Pereira Taranto

**Affiliations:** ^1^Mauá Institute of Technology (IMT), Praça Mauá 1, 09580-900 São Caetano do Sul, SP, Brazil; ^2^School of Chemical Engineering, University of Campinas (UNICAMP), Avenue Albert Einstein 500, 13083-852 Campinas, SP, Brazil

## Abstract

The objective of this work was to study the coating process of nifedipine extended release pellets using Opadry and Opadry II, in a fluid bed coater with a Wurster insert. The coating process was studied using a complete experimental design of two factors at two levels for each polymer. The variables studied were the inlet air temperature and the coating suspension flow rate. The agglomerate fraction and coating efficiency were the analyzed response variables. The air temperature was the variable that most influenced the coating efficiency for both polymers. In addition, a study of the dissolution profiles of coated and uncoated pellets using 0.5% sodium lauryl sulfate in simulated gastric fluid without enzymes (pH 1.2) was conducted. The results showed a prolonged release profile for the coated and uncoated pellets that was very similar to the standards established by the U.S. Pharmacopoeia. The drug content and the release profiles were not significantly affected by storage at 40°C and 75% relative humidity. However, when exposed to direct sunlight and fluorescent light (light from fluorescent bulbs), the coated pellets lost only 5% of the drug content, while the uncoated ones lost more than 35%; furthermore, the dissolution profile of the uncoated pellets was faster.

## 1. Introduction

The administration of drugs by oral dosage is the most typical, comfortable, and convenient way to release an active substance in an organism. Among the various pharmaceutical forms in which active substance release systems can be designed for oral use, pellets have attracted increasing interest due to several technological and therapeutic advantages [1–4]. Pellets have excellent flow properties, mainly due to their spherical shape, narrow particle size distribution, and surface susceptibility to film coating for the purpose of enteric protection or extended release.

The technique used to manufacture pellets is extrusion and spheronization. This process was first reported for use as a pharmaceutical application by two classic papers in 1970 [1, 5]. Although the extrusion/spheronization technique creates spherical granules as a product, it differs from the granulation technique concerning the wet weight treatment of the fine powders, as well in as the equipment used. The extrusion/spheronization technique is composed of four unit operations: granulation, extrusion, spheronization, and drying.

The pellets are ideal for the application of coatings due to their spherical shape. The film coating application for pharmaceutical use may be chosen for functional or esthetic reasons. The functional objective of film coating is to form a barrier that protects the pellets from the environmental conditions and/or to modify the drug release profile.

Fluidized beds are widely used in the pharmaceutical industry for coating solid particles such as pellets, granules, and powders. Initially, the particles are fluidized by hot air, and the coating solution or suspension is sprayed over the particles. Due to the hot air, the solvent evaporates and forms a solid film that surrounds the core material. The main challenge of this process is to form a uniform and continuous film coating on the pellet surface. The complexity lies in the large number of variables involved in the process, which makes studying this coating process relevant to the pharmaceutical industry [6]. The Wurster apparatus [7] is considered the most useful equipment for small-particle film coating [8].

Teunou and Poncelet [9] conducted a review of coating in fluidized beds and showed that the Wurster fluidized bed is the most suitable system for particle coating. They described the coating process in fluid bed with a Wurster insert and showed that to achieve an excellent coating, the particles must be dry during the ascent path because agglomeration will occur if the particles become wet in the annular region. This agglomeration occurs because the particles in the annular region are very close to each other, and the air velocity in this region is very low compared with the velocity in the region of the inner tube.

Albanez et al. [10] studied the process of coating diclofenac sodium pellets produced by extrusion/spheronization with an enteric release polymer (Acryl-Eze MP) in a Wurster fluidized bed. They studied the influence of two process variables: the inlet air temperature and the suspension flow rate. The evaluated responses were the efficiency of the coating process and the agglomeration index. In all tests, the coating efficiency exceeded 70%. It was concluded that both the inlet air temperature and suspension flow rate significantly (95% confidence level) influenced the coating efficiency and the agglomeration index. Only the interaction between the variables had no influence on the responses analyzed. A higher suspension flow rate improved the coating efficiency; however, it favored agglomeration. On the other hand, a higher inlet air temperature also led to agglomeration, which was not expected and may be explained by the influence of temperature on the adhesion of the film coating. The drug content and the release profiles were not significantly affected by storage at 40°C and 75% relative humidity.

Currently, the amount of commercially available polymeric suspensions and the variety of different required release profiles are very large. Polymeric suspensions are very well accepted by the pharmaceutical industry because the suspensions are easy to prepare and are of low cost. Among commercialized suspensions, aqueous forms are preferred because they cause less damage to the environment and do not pose poisoning risks.

Nifedipine is an active ingredient that is poorly soluble in water and is widely used as a calcium-blocking agent whose efficacy and tolerability have been demonstrated in numerous studies [11]. When exposed to daylight or certain wavelengths of artificial light, nifedipine is converted to the derivative nitrosophenylpyridine. The exposure of nifedipine to UV light leads to the formation of the derivative nitrophenylpyridine [12]. The pharmacokinetics and pharmacodynamics of nifedipine have been characterized using several drug formulations intended for both oral and parenteral use. It has been shown that the fast increase of nifedipine concentration in plasma results in acceleration of the heart rate and side effects [13–15]. Therefore, modified release formulations of nifedipine are the preferred therapeutic choices.

Due to its short half-life in vivo, immediate release doses of nifedipine should be given three times a day [16]. This therapeutic regime causes fluctuations in plasma levels, which are responsible for side effects. For this reason, it is appropriate to develop controlled release formulations that promote adherence to treatment and reduce undesirable effects [16, 17].

The development of controlled release forms is hampered by the low solubility of the molecule, which affects its absorption rate. Measures such as particle size reduction and polymer solid dispersion [16, 18] have been proposed as ways to increase the drug's bioavailability.

Considering all the above-mentioned advantages of multiparticulate systems and the need to protect nifedipine from light exposure, the purpose of this work was to develop nifedipine extended release multiparticulates produced by the extrusion/spheronization process [19] and coat them with different commercial powders (Opadry and Opadry II). These polymers were chosen because they contain titanium dioxide in their formulation and can protect the microgranules from light exposure. The influence of the inlet air temperature and the suspension flow rate on the coating process was evaluated, and the surface responses to coating efficiency and the agglomerate fraction were investigated. The drug release profile is in accord with that established in the United States Pharmacopeia [12].

## 2. Materials and Methods

### 2.1. Chemicals

Nifedipine was manufactured by Asmidhi Labs (India). Microcrystalline cellulose (MCC) 101, the main diluent in pellet manufacture, was obtained from Mingtai Chemical (Taoyuan Hsien, Taiwan). Lactose, used as a diluent, and polyvinylpyrrolidone (PVP-K30), used as a binder, were manufactured by Valdequímica Produtos Químicos Ltda (Brazil). Croscarmellose sodium manufactured by Amishi Drugs and Chemicals (Ahmedabad, Gujarat, India) was used as a disintegrant. Polyethylene glycol (PEG4000), used as a plasticizer and a lubricant, was manufactured by* Valdequímica Produtos Químicos Ltda* (Brazil). Methocel was manufactured by Colorcon (UK); it was used as a binder and was also added in a 1% w/w aqueous solution as the granulation liquid. Silicon dioxide was used as an adsorbent; it was manufactured by Longyan Shenghe Trading (German). The polymers used to coat the pellets were Opadry and Opadry II, which were manufactured by Colorcon (Dartford, Kent, UK). Opadry contains mostly hydroxypropyl methylcellulose, while Opadry II contains mostly polyvinyl alcohol.

### 2.2. Equipment

The blending and granulation were performed in a planetary mixer. To extrude the dough, a roller extruder (model EX50, Zelus, São Paulo, Brazil) with a 1.0 mm screen was used at 50 rpm. For the spheronization step following the extrusion, a spheronizer was used (model ES-015, Zelus, Sao Paulo, Brazil) with a rotation velocity of 900 rpm and perpendicular-type spheronization plate grooves with a diameter of 23 cm. An oven with forced air circulation and temperature control (model 420-4D, Nova Ética, São Paulo, Brazil) was used to dry the pellets. A screen pack with steel screens with openings between 0.425 mm and 1.40 mm was used for particle size classification of the pellets. A UV/VIS spectrophotometer (Cary, Varian, USA) was used to determine the drug content of the pellets and the amount of released drug in the in vitro dissolution tests. A scanning electron microscope (LEO 440, Campinas, Brazil) was used for the morphological analysis of the pellets. In the dissolution tests, a dissolver (model 299, Nova Ética, São Paulo, Brazil) with 6 tanks, each with a capacity of 900 mL and temperature and rotation control, was used. The film coating was performed in a fluid bed coater with a Wurster insert, (model R-060, by Zelus, São Paulo, Brazil).

### 2.3. Preparation of Extended Release Pellets

The pellets were prepared by the extrusion/spheronization process. The mixing of the powders and addition of a 1% w/w methanol aqueous solution were performed in a planetary mixer. The wet mass was passed through a gravity feed lab-scale radial extruder immediately thereafter. Batches of 270 g were spheronized at 900 rpm for 40 seconds in a lab-scale spheronizer. The pellets were dried in a hot air oven at 50°C for 24 h. The formulation that was tested is shown in Table 1.

### 2.4. Film Coating

**Figure 1:**
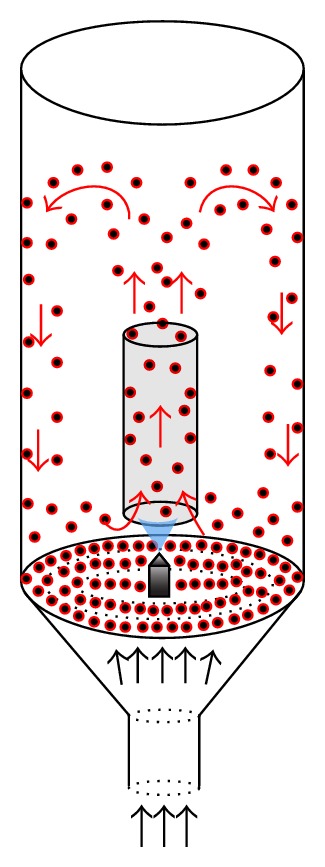
Schematic representation of the Wurster process.

In the Wurster process, a coating solution is sprayed on a particle bed moved by an ascending gas stream. The solution coats the particle in a simultaneous process of wetting and drying to form a layer with specific characteristics (Figure 1). The coating experiments were performed in a fluid bed coater column with a Wurster insert. The main parts of the fluidized bed used in this work are a conical base (top diameter: 135 mm, bottom diameter: 77.5 mm), an air distribution plate, a draft tube (height: 153.5 mm, inner diameter: 33 mm, gap from the bottom: 7.0 mm), a cylindrical glass vessel (inner diameter: 140 mm) and a double-fluid nozzle with external mixing (orifice diameter: 0.7 mm).

The airflow rate used in the tests was 1.9 × 10^−2^ kg/s, 1.15 times higher than that of minimum fluidization, as the pellets produced were Geldart's group D (density: 1455 kg/m^3^ and medium diameter: 1.04 × 10^−3^ m) particles. The initial mass of pellets was 350 g, with the size distribution shown in Table 2. A double-fluid atomizing nozzle with an orifice of 0.7 mm was used. The atomizing air absolute pressure was 2.0 bar. The coating suspension was kept under agitation during the coating experiments while being fed with a peristaltic pump (Provitec, DM7900, São Paulo, Brazil). After the suspension flow was stopped, the pellets remained in the cyclic bed for 5 min. The moisture content of the coated and uncoated pellets was determined using an oven with forced air circulation and temperature control until a constant mass (50°C for 48 h) was reached.

A two-level factorial design was performed for each polymer to identify the influential variables in the coating process, which are inlet air temperature (55 and 65°C) and suspension flow rate (5.53 and 6.64 g/min for Opadry; 5.37 and 6.46 g/min for Opadry II), in the coating process. This design determines which factors have important effects on the response as well as how the effect of one factor varies with the level of the other factors. Three runs were performed at the central point (60°C and 6.09 g/min for Opadry; 60°C and 5.92 g/min, Opadry II). The response variables were the coating efficiency and the agglomerate fraction, which are defined as follows. The coating efficiency (*η*) was calculated by dividing the actual mass gain by the theoretical mass gain. The actual mass gain (*φ*) was determined by weighing the dried pellets before and after coating, and the theoretical mass gain is the gain that would have been achieved if all of the solid material in the suspension had adhered to the surface. The agglomerate fraction (*f*_agg_) is given by the mass of agglomerates in relation to the total mass of coated pellets. Particles larger than 1.40 mm were considered agglomerates. Statistical analyses were performed using Statistica 10.0 software. The analysis of variance and the graph of the values predicted by observation were analyzed, and the response surfaces for the coating efficiency and agglomerate fraction were traced.

As shown in Table 1, the extended release pellets produced were coated with the aim of protecting them from exposure to light without changing the dissolution profile because the polymers used are for immediate release. The theoretical weight gain in the coating process was approximately 11%, as shown in Table 3.

### 2.5. Drug Content

The drug content of both coated and uncoated pellets was determined by powdering 300 mg of the pellets. The drug was then extracted with a methanol solution. The filtered extract was assayed spectrophotometrically at a wavelength of 350 nm (according to graphs of the absorbance spectrum and information obtained from the USP XXXII). The drug content determination was performed in triplicate, and all tests were performed in the absence of light and using glassware wrapped in aluminum foil.

### 2.6. Dissolution Tests

Both uncoated and coated pellets were subjected to dissolution studies to verify the extended release profile. In this analysis, 0.5% sodium lauryl sulfate in simulated gastric fluid without enzymes (pH 1.2) at 37°C was used as the dissolution medium for 12 h. Apparatus 1 (basket) was used at 100 rpm. Two replicate samples of approximately 50 mg of particles were put in the baskets. A sample of 5 mL from each vessel was filtered using a 0.45 *μ*m filter (Sartorius, Minisart RC25), and the dissolved amount of nifedipine was assayed spectrophotometrically at wavelength of 238 nm (according to graphs of the absorbance spectrum and information obtained from the USP XXXII). The rotation speed was 100 rpm. The dissolution tests were performed in a 6-vessel dissolver (Nova Ética, Brazil).

### 2.7. The Coating Suspensions

Opadry and Opadry II are fully formulated dry coating systems that are dispersible in water and use (hydroxypropyl methylcellulose) HPMC and (polyvinyl alcohol) PVA, respectively, in their formulations. These polymers contain titanium dioxide in their formulations, which may protect the microgranules from exposure to light, thus avoiding drug degradation. The suspension containing Opadry was prepared with 12% w/w of powder dispersed in water, and the suspension containing Opadry II was prepared with 20% w/w powder in water. The rheology of the coating suspensions was determined using a Brookfield Rheometer.

### 2.8. Scanning Electron Microscopy (SEM)

The particles were subjected to scanning electron microscopy with an LEO 440 Stereoscan microscope. This analysis aimed to visualize the surface morphology. The samples were mounted onto circular aluminum stubs with double-sided carbon tape and then coated with platinum.

### 2.9. Storage Stability

For a commercial product, the guarantee of stability is vital for its safety and efficacy during storage and use. In this study, coated and uncoated pellets were stored under stress conditions of 40°C and 75% relative humidity. The drug content and dissolution profiles were measured after 30, 60, 90, and 180 days.

For the photostability study, the samples were exposed to a fluorescent light and daylight for ten days. The drug content and dissolution profiles were measured.

## 3. Results and Discussion

### 3.1. Coating Suspension Rheology

**Figure 2:**
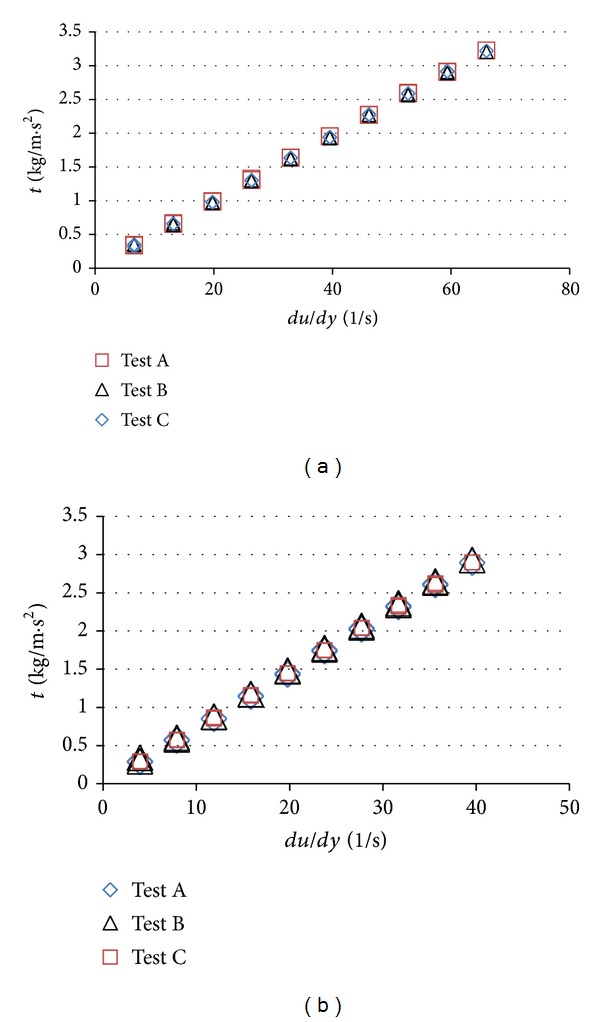
Experimental curves forshear stress versus deformation rate (Opadry II (a) and Opadry (b)).

The polymeric suspensions were prepared at the maximum concentration following the manufacturer's indications: 12% w/w Opadry and 20% w/w Opadry II. For both coating suspensions (Opadry and Opadry II), the shear stress varies linearly with the deformation rate; in other words, the shear stress is directly proportional to the velocity gradient. Therefore, the two polymeric suspensions, Opadry II and Opadry behave as in the model proposed by Isaac Newton and are thus Newtonian fluids. The experimental curves for shear stress and viscosity are shown in Figure 2 and Table 4, respectively.

The viscosity of the polymeric suspension of Opadry II is much lower than that of the Opadry suspension, although its solid content is higher. The low viscosity of Opadry II results in excellent and uniform droplet size, improving its performance in the coating process when compared with that of Opadry, which presented higher viscosity values (Table 4).

### 3.2. Uncoated Pellet Dissolution Studies

**Figure 3:**
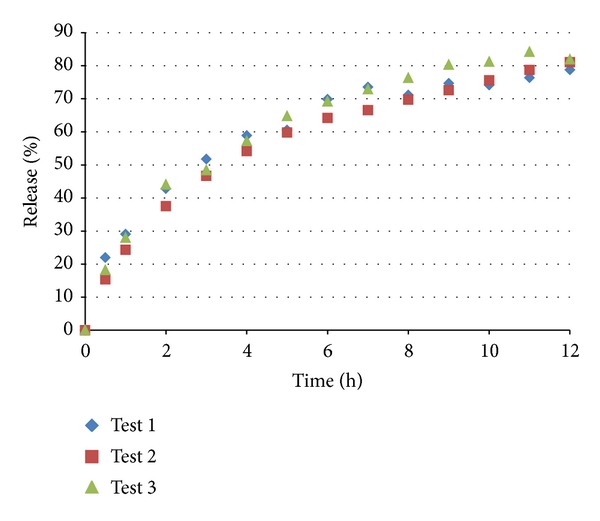
Dissolution profile of uncoated pellets in 0.5% sodium lauryl sulfate in simulated gastric fluid without enzymes (pH 1.2).

Dissolution tests were performed with the uncoated pellets according to the methods described in Section 2.6. As shown in Figure 3 and Table 5, the uncoated pellets presented an extended release profile that is similar to the United States Pharmacopeia [12] standards. The drug release profile for the nifedipine pellets was controlled by varying the ratio of microcrystalline cellulose, croscarmellose sodium, and lactose in the pellet formulation combined with the use of the controlled release polymers, as shown in Table 1.

### 3.3. Coating Process

**Figure 4:**
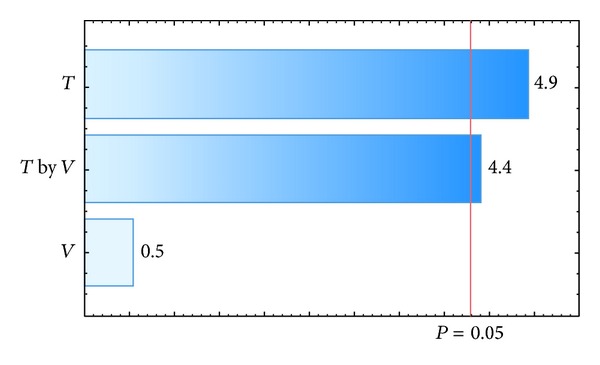
Pareto chart of effects: coating efficiency, *η-*Opadry.

**Figure 5:**
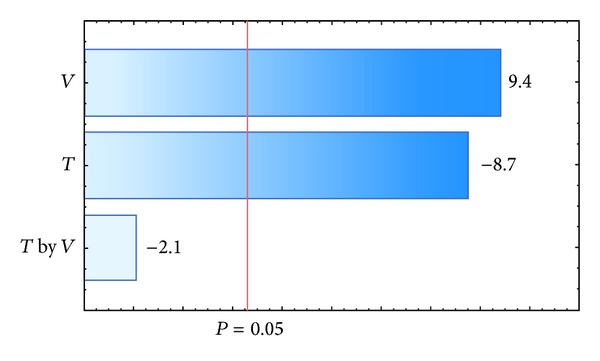
Pareto chart of effects: agglomerate fraction, *f*_agg_-Opadry.

**Figure 6:**
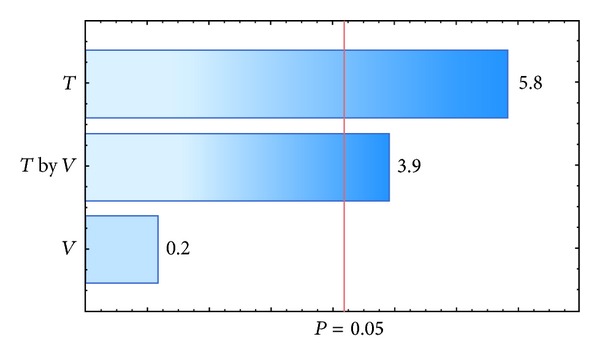
Pareto chart of effects: coating efficiency, *η-*Opadry II.

**Figure 7:**
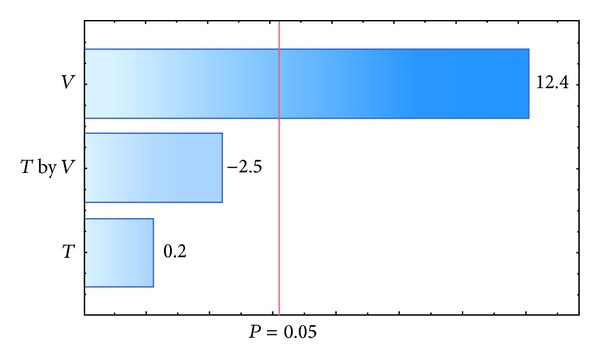
Pareto chart of effects: agglomeration fraction, *f*_agg_-Opadry II.

Coating experiments were performed for each polymer (Opadry and Opadry II) using a 2^2^ factorial design. The aim of this analysis was to investigate the influence of process variables on the coating performance and was determined by the two response variables *η* and *f*_agg_. The results for each polymer investigated are presented in Table 6. The response variable *η* was affected by inlet air temperature, the interaction between inlet air temperature, and the suspension flow rate for a *P* value of 0.05, as shown in the Pareto charts in Figures 4 and 6. The values that appear above the bars in Figures 4, 5, 6, and 7 are the calculated effects whose level of significance must be compared with the *P* value of 0.05 [20].

The variable that most influenced the coating efficiency was the air temperature. The coating performed at higher temperatures and flow rates (test 2; Table 6) resulted in improved coating efficiency, and the efficiency of Opadry II was very close to 100%. The lowest coating efficiency was obtained at a low air temperature and high flow rate (test 1; Table 6); this occurred because the air temperature was not sufficient to evaporate the solvent adhered to the pellets, which increased the agglomerate fraction.

The suspension flow rate was the only factor that significantly influenced the agglomerate fraction when the pellets were coated with a polymeric suspension of Opadry II (Figure 7). The positive effect of this factor showed that a higher flow rate resulted in an increased agglomerate fraction, which is undesirable in the coating process and corroborates the observations by Albanez et al. [10] in their coating process study of diclofenac sodium pellets. The reason for this behavior is that the drying did not occur fast enough to dry the suspension in the spout region of the bed, and the wet pellet surface caused the adhesion of the particles. In addition to the suspension flow rate, the inlet air temperature also significantly influenced the agglomerate fraction when the coating process was carried out with the polymeric suspension of Opadry (Figure 5). The negative effect of inlet air temperature meant that when the coating process was carried out at lower air temperatures, a higher agglomerate fraction resulted. Because drying occurs faster at higher temperatures, the increase in agglomeration can be explained by a greater amount of liquid on the surface.

**Figure 8:**
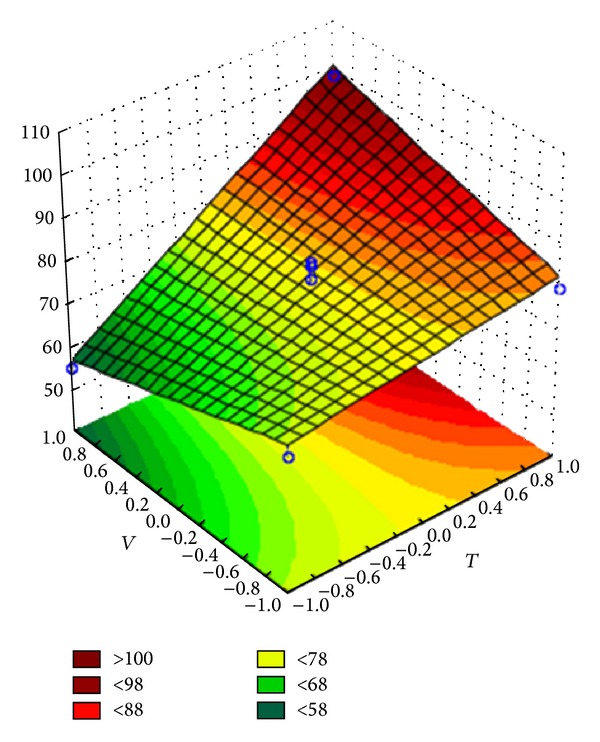
Response surface for the coating efficiency of Opadry II.

**Figure 9:**
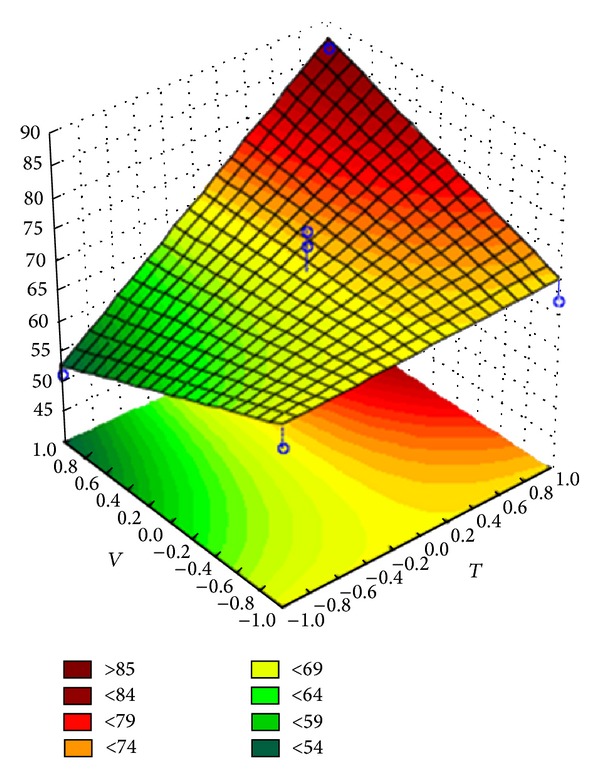
Response surface for the coating efficiency of Opadry.

**Figure 10:**
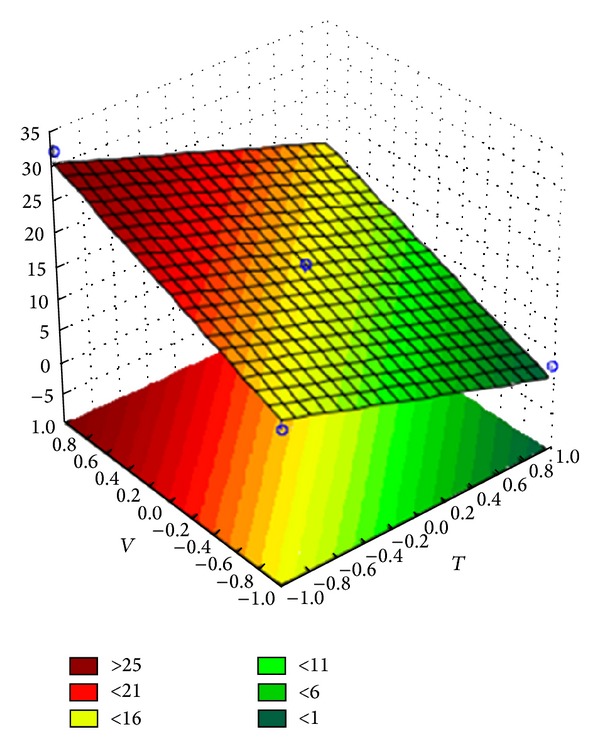
Response surface for the agglomerate fraction of Opadry.

The variance analysis showed that the regression is significant for the predictive linear models of the coating efficiency (for both Opadry and Opadry II) and the agglomerate fraction (only for Opadry). The response surfaces of the predictive models for coating efficiency are shown in Figures 8 and 9, and the equations of the linear models are shown in coded variables in (1) and (2). The response surface of the predictive model for the agglomerate fraction is shown in Figure 10, and the equation of the linear models is shown in coded variables in (3):

(1) η  (T,V)=78.690+12.800·T+8.610·T·V

(2) η  (T,V)=70.610+9.293·T+8.273·T·V

(3) fagg(T,V)=15.464−7.265·T+7.625·V.

The response surfaces indicate that elevated inlet air temperature and flow rate increase the efficiency of the coating process; however, an elevated suspension flow rate increases the agglomerate fraction during the coating process.

The suspension of Opadry II was favorable for increased coating efficiency, although its solid content was greater than that of the suspension of Opadry, because the suspension containing Opadry II had a much lower viscosity than the suspension containing Opadry; it also contained talc as a surfactant agent, which reduced the surface tension, promoting the spread of the suspension over the particle surface and improving its wettability. Moreover, the coating time with the Opadry II polymeric suspension is significantly lower than the process time of the Opadry polymeric suspension for the same mass gain.

The amount of the suspensions sprayed onto the pellets was sufficient for theoretical mass gains of 11%. This value was recommended by the manufacturer. The actual mass gains from the tests are shown in Table 7.

**Figure 11:**
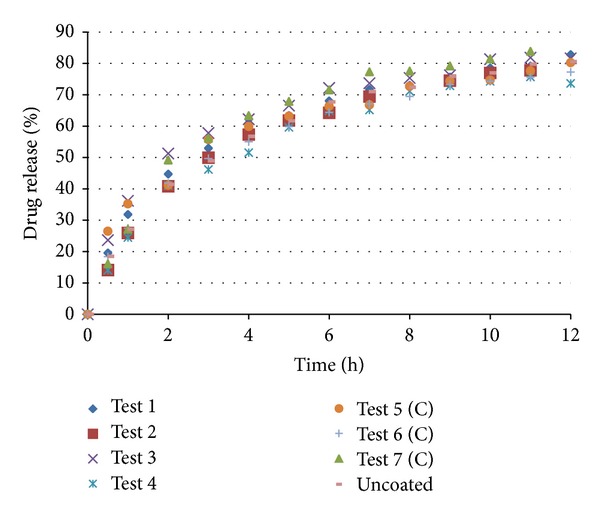
Dissolution of pellets coated with Opadry II.

**Figure 12:**
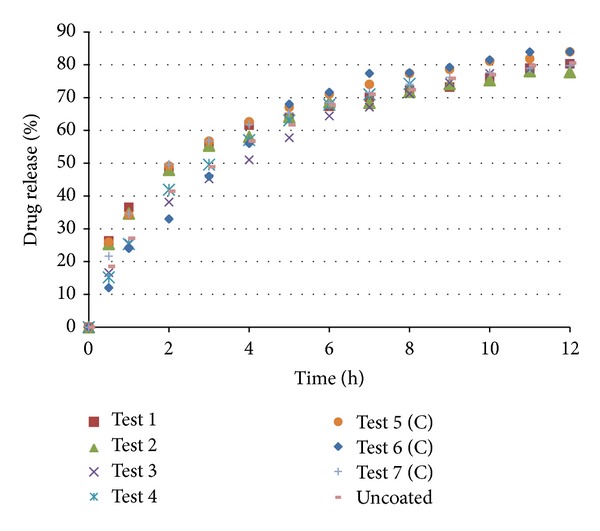
Dissolution of pellets coated with Opadry.

The coated pellets were submitted to in vitro dissolution tests. The results are shown in Figures 11 and 12. The coated pellets presented release profiles similar to those of uncoated pellets. This result was expected because the polymers applied in the coating process are immediate release, not modified release. Therefore, the coated pellets presented extended release profiles that were similar to those of the United States Pharmacopeia [12] standards (Table 5).

**Figure 13:**
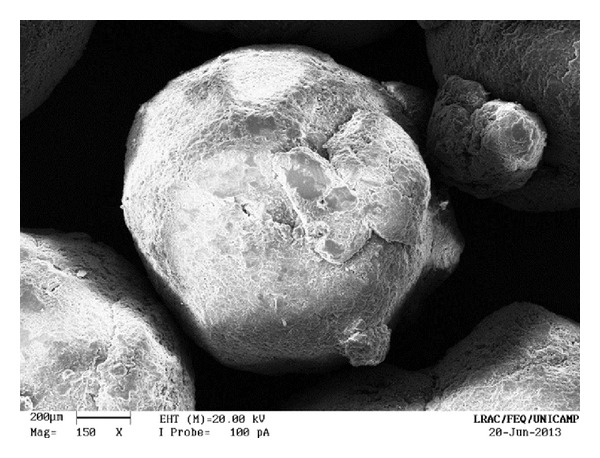
Uncoated pellets (150x).

**Figure 14:**
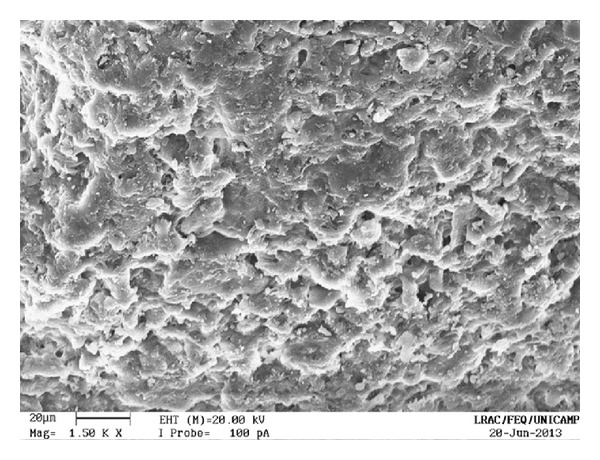
Uncoated pellets (1500x).

**Figure 15:**
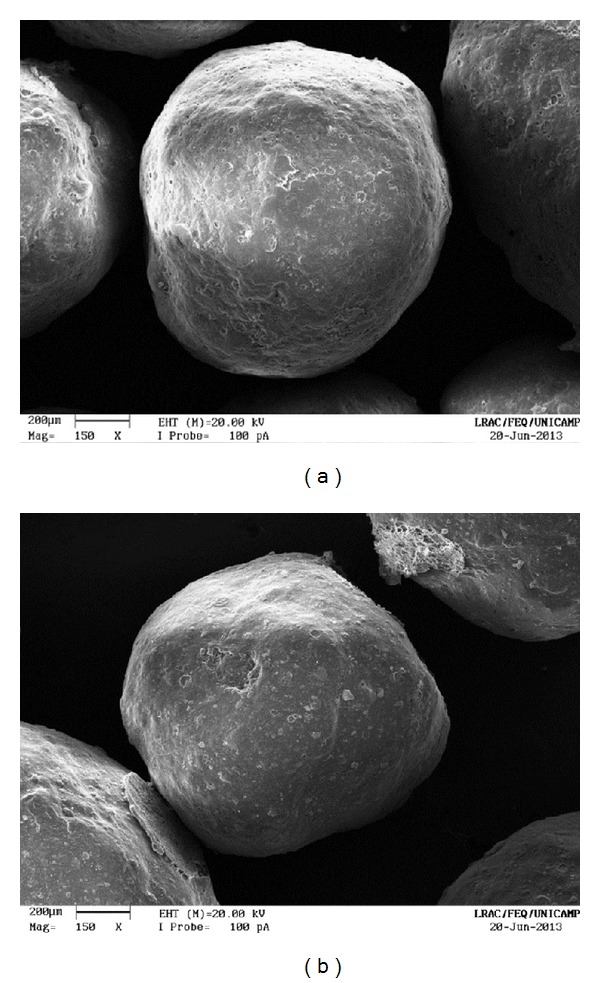
Coated pellets (150x): (a) Opadry II; (b) Opadry.

**Figure 16:**
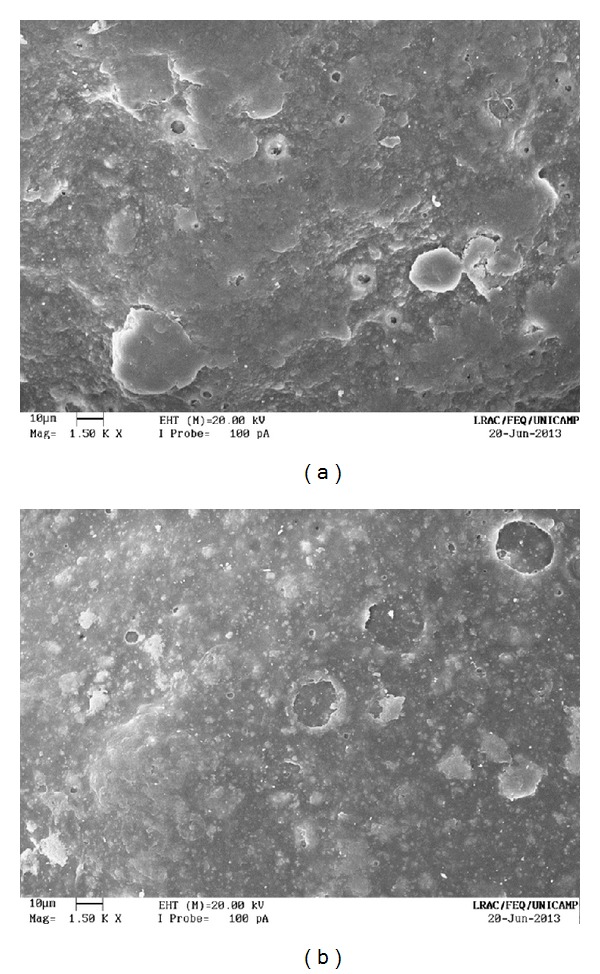
Coated pellets (1500x): (a) Opadry II; (b) Opadry.

**Figure 17:**
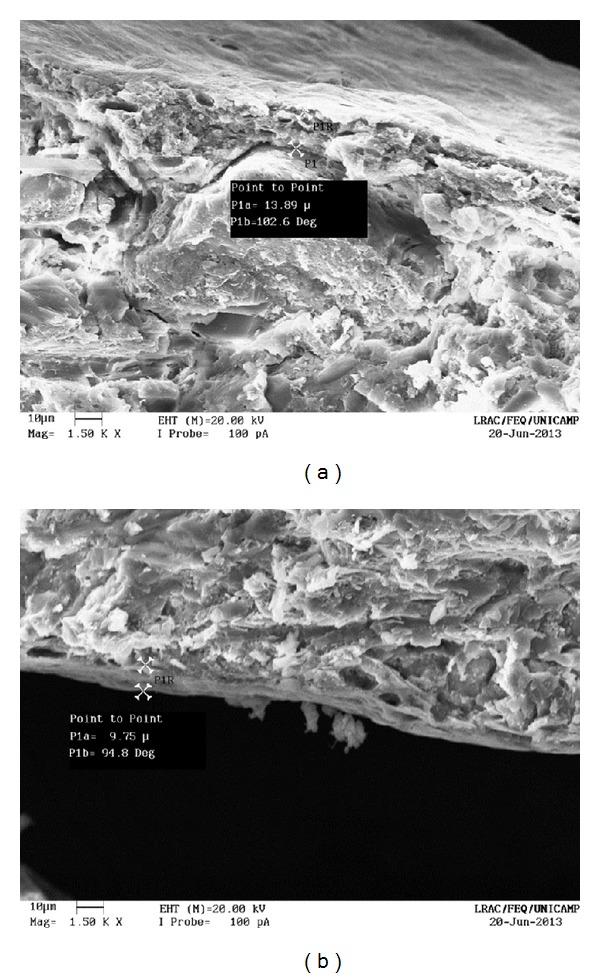
Coated pellets (1500x).

The uncoated pellets have a rough, irregular surface, as shown in Figures 13 and 14. When the polymer coats the granule, the surface is smoothened and rounded, with no visible cracks. As a consequence, the film creates a barrier between the environment and the pellet. The surface of a coated particle is shown in Figures 15 and 16. In Figure 17, coating layers of approximately 14 *μ*m and 10 *μ*m are visible on pellets coated with Opadry II and Opadry, respectively. These pellets were obtained with a 9.43% and 8.54% mass gain (test 5 (C)—Table 7) using polymeric suspensions of Opadry II and Opadry, respectively.

### 3.4. Stability

**Figure 18:**
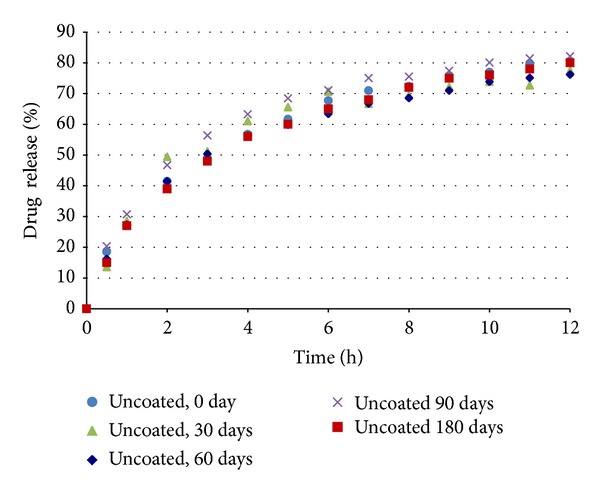
Dissolution profiles of uncoated pellets before and after storage under stress conditions.

**Figure 19:**
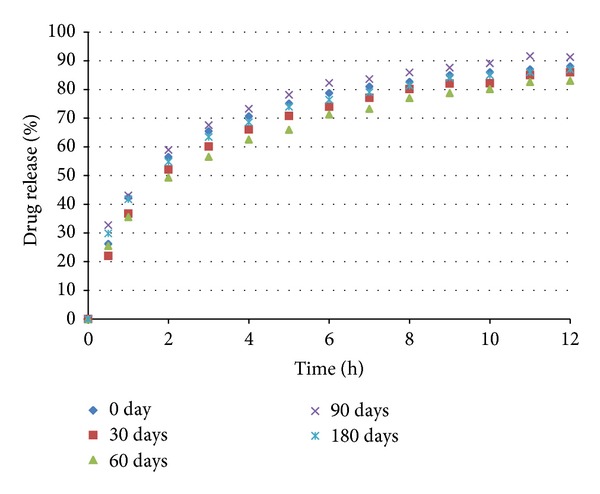
Dissolution profiles of pellets coated with Opadry II (test 5 (C)) before and after storage under stress conditions.

**Figure 20:**
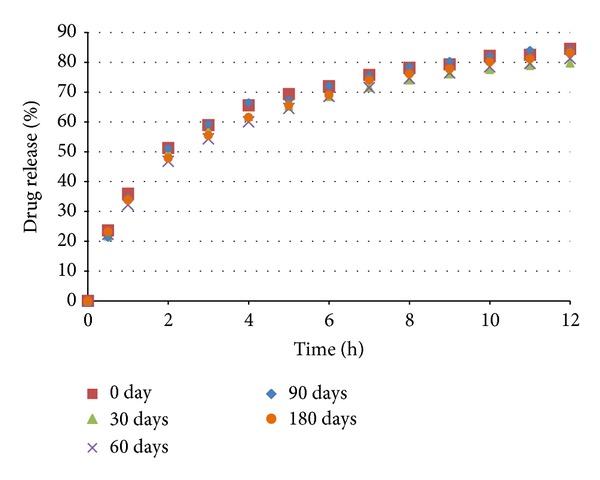
Dissolution profiles of pellets coated with Opadry (test 5 (C)) before and after storage under stress conditions.

**Figure 21:**
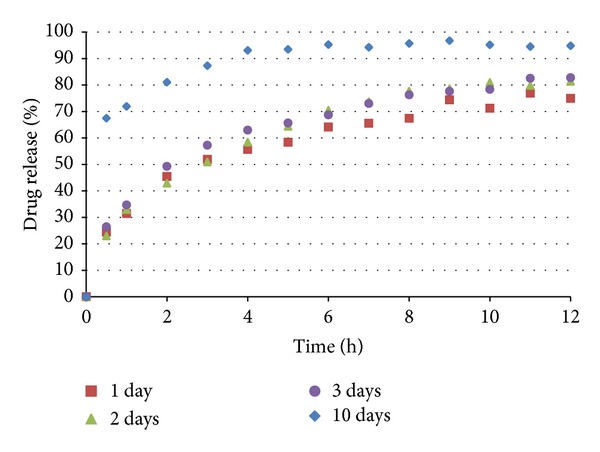
Dissolution profiles of uncoated pellets after storage under light stress conditions.

**Figure 22:**
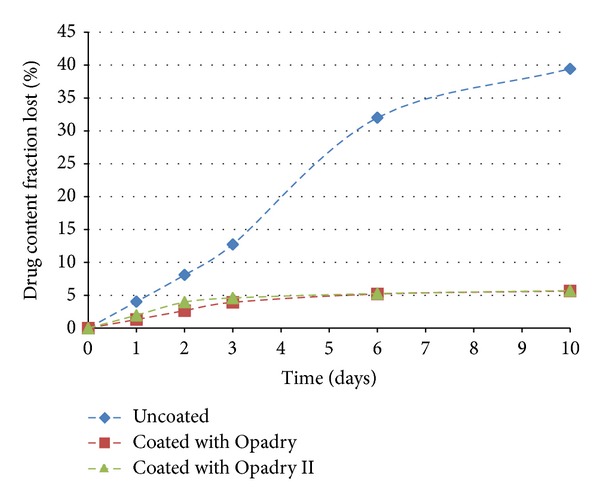
Drug content fraction lost after storage under light stress conditions.

The accelerated stability tests (40°C, 75% rh) were performed as described in Section 2.9. In Figures 18, 19, and 20 the dissolution profiles of the uncoated and coated pellets for the 180 study days can be observed. The coated and uncoated pellets remained stable. The amounts of drug released at 30, 60, 90, and 180 days were very similar to the amount released at the beginning (day 0), as discussed in Section 3.2. Furthermore, the drug content of the pellets remained constant during this period. These results indicate that during the period of 180 days, there was not sufficient physical and chemical degradation in the pellet matrix to modify the drug dissolution profile. However, when the pellets were subjected to light stress conditions, only the drug content and dissolution profile of the uncoated pellets were changed, as shown in Table 8 and Figure 21. In the uncoated pellets, the nifedipine degraded at a rate much faster than in the pellets coated with either of the two polymeric suspensions. After 10 days of light exposure, the coated pellets had lost only approximately 5% of the drug content, while the uncoated pellets had lost 40%.  Figure 22 shows the fraction of drug content lost from the coated and uncoated pellets during the 10 days of light exposure. Therefore, the coating of nifedipine extended release pellets is necessary to protect them from light, thereby avoiding drug degradation and changes in the dissolution profile. The two polymeric suspensions used for coating the pellets contain titanium dioxide, which acts as an opacifying agent, protecting the pellets from exposure to light and increasing their shelf life; this corroborates the reports of Rowe et al. [21] and Rocha and Taranto [22].

## 4. Conclusions

The statistical analysis of the coating process showed that the inlet air temperature and the interaction between the air temperature and the suspension flow rate influenced the coating efficiency with a 95% confidence level. The variable that most influenced the coating efficiency was the inlet air temperature. Higher suspension flow rates resulted in better coating efficiency but also favored agglomeration, which was expected. The coating performed at higher temperatures and flow rates resulted in improved processing efficiency, and the efficiency of the Opadry II polymer was very close to 100%. The coating time with the polymeric suspension of Opadry II was significantly lower compared to the processing time for the polymeric suspension of Opadry for the same mass gain. The suspension flow rate was the only factor that significantly influenced the agglomerate fraction when the pellets were coated with a polymeric suspension of Opadry II. In addition to the suspension flow rate, the inlet air temperature also significantly influenced the agglomerate fraction when the coating process was carried out with a polymeric suspension of Opadry. The response surfaces indicate that the path of maximum slope (higher agglomerate fraction) occurs when the coating process is carried out at high flow rates and low air temperatures. The in vitro dissolution studies showed that the nifedipine release profile was not affected by the polymeric coatings. However, the photostability studies showed that the coating of nifedipine extended release pellets is necessary because the dissolution profile and drug content were significantly altered for only the uncoated pellets when they were exposed to light.

## Figures and Tables

**Table 1:** Powder mass fractions used in the preparation of extended release nifedipine pellets.

Material	w/w (%)
PEG4000	15.0
Microcrystalline cellulose (MCC)	26.5
Lactose	26.5
Croscarmellose sodium	2.0
Methocel	1.0
PVP-K30	4.0
Nifedipine	25

**Table 2:** Size distribution of the pellets used in the coating experiments.

Size range (mm)	Opadry II		Opadry	
	*M* (g)	%	*M* (g)	%
1.18 < *d* < 1.40	67	19	82	24
1.00 < *d* < 1.18	154	44	146	42
0.85 < *d* < 1.00	95	27	87	25
0.71 < *d* < 0.85	34	10	35	10

Total	350	100	350	100

**Table 3:** Theoretical weight gain in the coating process.

Test	Opadry II	Opadry
	ϕ (%)	ϕ (%)
1	7.4	10.5
2	10.8	11.9
3	10.8	11.7
4	10.9	11.7
5(C)	11.4	11.4
6(C)	11.2	9.3
7(C)	11.0	10.6

**Table 4:** Viscosity values of coating suspensions.

Opadry II		Opadry	
Test	µ (kg/m·s)	Test	µ (kg/m·s)
A	0.0486	A	0.0735
B	0.0484	B	0.0737
C	0.0488	C	0.0735
Mean	0.0486 ± 0.00014	Mean	0.0736 ± 0.00010

**Table 5:** Absolute values of the average fraction of nifedipine released in the in vitro dissolution tests of the pellets and the value ranges established by the US Pharmacopoeia.

Time (h)	Amount dissolved (%), experimental	Amount dissolved (%), Pharmacopeia
1	27.1 ± 2.5	10–35
4	56.7 ± 2.4	40–67
12	80.5 ± 1.6	Not less than 80

**Table 6:** Influence of process variables on coating performance with the different polymers.

Test	*T*	*V*	Opadry II		Opadry	
			*f*_agg_ (%)	*η* (%)	*f*_agg_ (%)	*η* (%)
1	−1	+1	13.9	55.4	32.7	51.5
2	+1	+1	12.2	98.2	14.4	86.7
3	−1	−1	2.8	71.8	13.7	66.1
4	+1	−1	4.8	80.2	2.9	68.1
5(C)	0	0	12.8	82.7	13.3	74.9
6(C)	0	0	11.6	83.2	14.6	77.2
7(C)	0	0	11.4	79.3	16.6	69.8

**Table 7:** Evaluation of coating experiments.

Test	*T*	*V*	Opadry II	Opadry
			*φ* (%)	*φ* (%)
1	−1	+1	4.1	5.4
2	+1	+1	10.6	10.3
3	−1	−1	7.8	7.7
4	+1	−1	8.8	7.9
5(C)	0	0	9.4	8.5
6(C)	0	0	9.3	7.2
7(C)	0	0	8.8	7.4

**Table 8:** Drug content with storage time under light stress.

Time (days)	Drug content of the pellets (%)		
	Uncoated	Opadry II	Opadry
0	24.4 ± 0.2	22.4 ± 0.3	22.1 ± 0.1
1	23.4 ± 0.2	21.9 ± 0.3	22.8 ± 0.2
2	22.4 ± 0.5	21.5 ± 0.1	21.6 ± 0.2
3	21.2 ± 0.4	21.4 ± 0.2	21.3 ± 0.3
6	16.6 ± 0.2	21.2 ± 0.2	21.0 ± 0.3
10	14.8 ± 0.1	21.1 ± 0.2	20.9 ± 0.2

## Nomenclature

*μ*: Viscosity (Pa · s)
*τ*: Shear stress (kg/m · s^2^)
*η*: Coating efficiency (%)
*φ*: Actual mass gain (%)
*ϕ*: Theoretical mass gain (%)
*f*_agg_: Agglomerate fraction (%)
*du*/*dy*: Deformation rate (1/s)
*V*: Flow rate (g/min)
*T*: Temperature (°C)
*M*: Mass (g).
